# “I need somebody who knows about feet” a qualitative study investigating the lived experiences of conservative treatment for patients with posterior tibial tendon dysfunction

**DOI:** 10.1186/s13047-019-0360-z

**Published:** 2019-11-07

**Authors:** Rona Frances Campbell, Christopher Morriss-Roberts, Beverley Durrant, Simon Cahill

**Affiliations:** 1 The London Podiatry Centre, 3 Courthill Road, London, SE13 6DN England; 2 University of Brighton, Leaf Hospital, St Anne’s Road, Eastbourne, BN21 2HW England; 3 University of Salford, Brian Blatchford Building, Salford, Manchester, M6 6PU England; 4 University of Brighton, Leaf Hospital, St Anne’s Road, Eastbourne, BN21 2HW England

**Keywords:** Posterior tibial tendon dysfunction, Qualitative research, Conservative treatment, Lived experience

## Abstract

**Background:**

Posterior tibial tendon dysfunction is a disabling, chronic, progressive tendon condition that detrimentally affects foot, ankle and lower limb function. Research suggests that posterior tibial tendon dysfunction is poorly recognised and difficult to treat. When posterior tibial tendon dysfunction is diagnosed, the clinician is faced with a weak evidence base and guidelines for the common conservative treatments.

to guide their management. Moreover, there are no current evidence-based guidelines for the conservative management of posterior tibial tendon dysfunction. Emerging research suggests that posterior tibial tendon dysfunction not only has a physical impact on the patient, but it also has psychosocial impact on quality of life.

Conservative treatments for posterior tibial tendon dysfunction are generally undertaken during early management. The most common are foot orthoses, exercises, bracing, lifestyle changes and injections. Quantitative evidence supporting conservative treatments for posterior tibial tendon dysfunction in relation to function, pain and patient reported outcome measures are reported in the literature.

There is a paucity of qualitative research investigating the psychosocial impact of the common treatments for posterior tibial tendon dysfunction. Interpretative phenomenology is concerned with lived experience which is involves the detailed exploration of experience which is embedded within the social and temporal contexts of the lifeworld of the person. The aim of study research is to investigate the lived experience of conservative treatments for patients who have posterior tibial tendon dysfunction using Interpretative Phenomenological Analysis.

**Methods:**

Five participants with posterior tibial tendon dysfunction were purposively recruited from a private podiatry practice and semi-structured interviews were conducted to examine their lived experiences of treatment for posterior tibial tendon dysfunction. The data for this study was collected and analysed using Interpretative Phenomenological Analysis.

**Results:**

This research identified three superordinate themes which influenced the lived experience of treatment for these patients (i) adverse experience during the patient journey (ii) treatment burden, and (iii) negative self-concept.

**Conclusion:**

This study highlights some of what is anecdotally known about the lived experience of treatment for patients with posterior tibial tendon dysfunction, but has never been studied in a qualitative, methodological manner. This study addresses the gap in the qualitative literature. It reveals novel aspects of the lived experience throughout the patient journey, the detrimental impact of treatment burden, loss and negative self-concept. This evidence is important because it highlights the need for a greater understanding of the psychological and social factors that can influence the lived experience of treatment for this group of patients.

## Background

Musculoskeletal (MSK) conditions encompass a spectrum of pathologies that affect muscles, soft tissues, joints and the spine [1]. MSK conditions are identified as the second highest cause of morbidity related global burden of disease and are the single biggest cause of growing burden of disability in the United Kingdom (UK). Evidence suggests that MSK conditions have the third worst quality of life (QoL) impact in the UK after neurological and mental health conditions [2, 3]. Effective management of many MSK conditions incorporates a spectrum of interventions including early diagnosis, assessment, optimal condition management and efficient service delivery. These tenants are set out in existing MSK frameworks and National Health Service (NHS) policies [2]. Early effective management of MSK conditions has been shown to help people stay in or return to work and carry out their social activities [3, 4].

Posterior tibial tendon dysfunction (PTTD) is a disabling, chronic, progressive, MSK condition typically with an insidious onset, that has a detrimental effect on lower limb function [5]. The pain, functional impact and disability that PTTD imposes on patients can influence QoL [6]. Data on prevalence is limited, however, it is estimated to occur in 10% of middle aged women within the UK population, but this estimate is likely to be higher as it can often be poorly recognised [7]. PTTD can also occur in athletic populations and in patients who have inflammatory arthritis [5].

PTTD can be classified into stages according to the clinical signs and progression of functional impairment [8–10]. These staged classifications, namely those based on the Johnson and Strom [10] classification, are widely referred to within clinical practice and the literature, despite a lack of information to substantiate their reliability or validity [11]. Evidence suggests that PTTD is poorly recognised by a range of practioners and is difficult to treat [12]. When patients are diagnosed they may not be referred on in a timely manner to practioners who can effectively manage PTTD [5, 6]. Often the most appropriate diagnostic imaging is not sought [12]. These issues may occur because there are no evidence-based guidelines for PTTD. Moreover, there is also a paucity of high quality evidence supporting conservative treatments [13]. This lack of guidance for practitioners, may hinder accurate, timely diagnosis and delay early effective conservative management which ideally should occur within the optimal therapeutic window [14].

Conservative treatments for PTTD include foot orthoses (FO), exercises, injections, bracing, footwear change, education regarding PTTD, disease self-management and lifestyle changes [15]. The quantitative efficacy of these treatments has been extensively researched [16–24]. The following studies included outcomes which investigated satisfaction and self-reported outcome measures. Patients with PTTD (*n* = 49) were provided with ankle foot orthoses (AFO) or a University of California Biomechanics Lab (UCBL) orthoses, and improvements were reported in pain, and function. Patient satisfaction was rated as excellent (67%), fair/poor (33%) and failed (9%) [25]. The authors state that there was a low uptake in surgery in this cohort and the reason given was ‘British stoicism’ [25]. There was no explanation as to what constituted fair/poor satisfaction or why 9% failed or the meaning of British stoicism. In a follow up study, 82% of patients categorised using a modified version of the Johnson and Strom classification (stages I-IV), reported they were satisfied with their conservative care. Participants received a UCBL device or ‘supportive’ accommodative orthoses or callipers (only in stage IV) and adjunctive treatments [26]. Poor results in satisfaction were reported within this cohort in stage III-IV participants and this was attributed to the age of the participants and their comorbidities. These are interesting findings which may have been better understood through qualitative research. Further research was carried out where participants (*n* = 47) with stage I & II PTTD were given UCBL orthoses if symptoms were present for < 3 months and an upright brace if symptoms were present for > 3 months [27]. Of this cohort, 89% had successful functional outcomes and all were ‘satisfied’. Two of the brace cohort who had minimal or no pain chose to continue with the brace and the orthotic patients reported that as symptoms reduced, orthoses became ‘unimportant’. Again, qualitative enquiry would have been useful to uncover the reasons behind these interesting outcomes. A study with the longest follow up (7–10 years) which investigated a brace cohort, reported 60% of patients were satisfied with the brace and treatment, however 35% were partially satisfied or unsatisfied with brace treatment [28]. The most recent randomised controlled trial in 2015 (*n* = 39) investigated the use of orthoses (off the shelf Airlift brace) augmented with either strengthening or stretching in patients with PTTD. All participants reported significant improvements in pain and disability but no improvements in self-reported outcome measures [19]. The greatest limitation in this body of literature is the lack of qualitative research which could unpack the reasons behind the nuances reported during treatments [12]. The lived experience is concerned with experiences and phenomena that are particular to a person and that hold significance to the person within their life [29, 30]. Studying this phenomena therefore provides the understanding of experience from those who have lived it [29]. Understanding the lived experiences for patients with PTTD may help to improve clinical outcomes for this group of patients in future. The aim of this study is to understand the lived experiences of patients undergoing treatment for PTTD.

## Methods

Interpretive phenomenological analysis (IPA) was used to collect, analyse and report the results [31]. IPA is centred in three keys areas of the philosophy of knowledge, phenomenology, hermeneutics and idiography [29]. Interpretative phenomenology is grounded in hermeneutics which is the theory of interpretation and thus enables the researcher to interpret the persons meanings and perspectives relative to the lived experience [29]. Through interpretation, the researcher is able to uncover the lived experience which is embedded within the participants lifeworld and their situated relationship to a specific phenomenon, in this instance the lived experience of treatments for PTTD [31, 32]. IPA differs from phenomenology alone as it is devoted to the study of experience, whereas IPA is also concerned with the interpretation and understanding of experience [29].

### Ethics

Ethical approval was sought and obtained from the University of Brighton, School Research Ethics and Governance Panel.

### Recruitment

Participants with PTTD were purposively recruited from a private podiatry clinic located in South-East London between February 2017–October 2017. Purposive selection was necessary to allow the researcher access to the experiences and patients related to the research question. Inclusion criteria specified any male or female participant diagnosed with PTTD aged between 18 and 80 years. Diagnosis was confirmed through clinical history, clinical examination and diagnostic ultrasound imaging. Exclusion criteria specified any patient with other diseases that may affect peripheral nerves, structure or function and pain perception, or participants that had incurred foot injury or had any surgical foot procedures. This was to ensure that the lived experiences of the treatments were related to PTTD alone and no other condition. Those with language barriers that would prevent them from giving informed consent were excluded.

### Participants

Participants were given a patient information sheet upon showing an interest in participating and were given the opportunity to contact the primary researcher (RFC) to ask questions. A member of the administration team contacted them 48 h after they received the information sheet and asked if they were willing to participate.

Those who agreed to participate were scheduled for an interview at their convenience. The aim was to recruit between four to six participants who had PTTD [33]. Five participants participated in the study. This sample size is justified to meet the obligations that are required of an IPA enquiry while being able to develop meaningful similarity and difference between cases [29]. Patient demographics, duration of symptoms, classification and details of clinical examination is summarised in Table 1.

**Table 1:** Patient demographics, classification and clinical examination on presentation to the private podiatry clinic

Participant (pseudonym)	Age	Gender	Duration of symptoms	Classification
Mark	76	Male	12 months	II-A2
Faye	80	Female	9 months	II-A2
Mila	40	Female	7 months	I-A
Stanley	60	Male	20 months	IV-A
Christopher	45	Male	14 months	II-B

Classification system of Bluman et al. [8].

Clinical examination:

• Clinical history

• Pain or swelling on palpation of the medial ankle

• Pain or weakness on the single heel raise test

• Assessment of foot posture

• Ultrasound imaging conducted to confirm diagnosis

Prior to all interviews and after written consent was obtained, RFC reiterated the points on the patient information sheet to ensure the participants were aware of the purpose of the study and how participating may impact upon them.

The participants were advised that pseudonyms would be allocated to the data to ensure anonymity and it was explained that data collected would be used for publication.

All signed consent forms are stored in a locked cabinet in one of the researcher’s university office (CMR) and will be kept for up to 10 years after the end of the study. All data collected is stored and secured under the requirements of the General Data Protection Regulations.

### Interviews

Interviews were conducted in a private room in the podiatry clinic, lasted approximately one hour and were digitally recorded [31]. A semi-structured interview schedule (Additional file 1) was used to facilitate a comfortable interaction with the participant. This comfortable interaction was established by asking ‘icebreaker’ questions prior to interview. Specific questions were asked and then prompts were used only when required during the interview. The interviews were conducted by RFC, who has previous experience in conducting semi-structured interviews and in IPA. RFC works in the private podiatry clinic where the participants were recruited. RFC is an experienced MSK podiatrist who worked in the NHS in multidisciplinary MSK teams for seven years and the last three years private practice. RFC has an MSc in Clinical Biomechanics and is working towards a qualification in diagnostic ultrasound. RFCs current caseload comprises of professional athletes, working as part of a surgical team, and managing complex biomechanical cases. This private podiatry clinic has a gait lab and part of RFCs clinical practice includes utilising F-Scan and Vicon motion technology as part of clinical assessment in managing complex biomechanical and surgical patients.

The schedule focused on the research question and was used to explore the lived experience for the participants [29]. The questions were devised through the researcher’s experience in treating PTTD. Questions were reviewed by CMR and then approved by the ethics committee of the University of Brighton. CMR has extensive practical and academic experience in IPA and is also a podiatrist.

Verbatim transcription and analysis of the data was completed by RFC. This is an important part of the methodological procedure as it immerses the researcher in the data during the analytic process [31].

### Analysis

There is no definitive method to follow when undertaking analysis, the founders of IPA suggest that a data analysis guide is used [32]. Data analysis in IPA is flexible and dynamic, the founders of IPA advocate an iterative approach to the data [29]. The iterative approach includes reading and re-reading the data, initial noting, developing emergent themes, searching for connections across emergent themes, moving to the next case and repeating this process on all transcripts [29]. The role of iteration in qualitative data analysis is analytical which means it can be repetitive, but is should not be a mechanical task. Iterative reflexivity is key in developing insight and unfurling perspectives while focusing on the meanings of the lived experience throughout the process of data analysis [32]. These steps were followed by RFC during analysis. Although, this was not a linear process because of the iterative and reflexive approach taken [31, 32]. During the process RFC sought feedback from co-authors and referenced field diaries to audit and ensure an agreeable triangulation of the themes that were emerging from the data during analysis.

## Results and discussion

Three superordinate themes emerged from the data and were categorised as: (i) adverse experience during the patient journey, (ii) treatment burden, and (iii) negative self-concept. Superordinate and subordinate themes can be found in Table 2.

**Table 2:** Table of themes

Superordinate themes	Subordinate themes
Adverse experience during the patient journey	Inappropriate referral Loss Stoicism
Treatment burden	Increased workload of healthcare
Negative self-concept	Metaphor and bracing Footwear and gendered embodiment Resistance to change

### Patient journey + inappropriate referral

The lived experiences of treatment for these participants was embedded within the social fabric of their lifeworld. As such, their experiences occurred over a continuum of time and within the context of their lives. Therefore, their journeys during treatment were a feature of their lived experiences. The participants accessed several healthcare services and treatments, prior to presenting at the private podiatry clinic (Table 3).

**Table 3:** Services accessed prior to presenting at private podiatry clinic

Participant (pseudonym)	General Practice	Orthopaedics	Physiotherapy	Osteopathy	Imaging
Mark	Yes	Yes	No	No	MRI
Faye	Yes	Yes	Yes	Yes	MRI
Mila	Yes	Yes	Yes	No	MRI
Stanley	Yes	Yes	Yes	No	DVT scan /MRI / US
Christopher	Yes	Yes	No	Yes	US

Abbreviations: *DVT* deep vein thrombosis, *MRI* magnetic resonance imaging, *US* ultrasound

Patients may describe their journeys through healthcare systems as complex, fragmented and frustrating [34]. Inadequate co-ordination of care can lead to negative outcomes and experience and may be due to inadequate clinical guidelines or due to referral processes [35]. The journey for participants in this study was influenced by inappropriate referral, length of their journey, and prolonged symptoms despite treatment. Participants revealed their experiences in terms of referral and the temporal aspects of their journey.

*“I was just feeling so desperate because it had been going on so long … I felt a bit lost … I think the hardest bit was I thought I was going to the right person, so I thought I was ok” (Mila).*

*“It had been going on so long … and there I was going to the fracture clinic with people in their arms in slings and you know in casts … I did not feel I was in the right place” (Christopher).*

Stanley experienced several unnecessary medical tests when the General Practitioner (GP) was trying to establish a diagnosis.

*“I had all of these blasted blood tests you know … and I went for a DVT scan” (Stanley).*

PTTD can be managed by a number of practitioners who have expertise in MSK injuries [12]. All the patients recruited into this study self-referred into the podiatry clinic after having has unsuccessful treatment elsewhere and were assessed by a podiatrist who had over 25 years in managing MSK injuries. Podiatrists that have expertise in managing MSK injuries are well placed to manage PTTD. Participants reported their lived experiences changed when they saw this podiatrist. However, the authors acknowledge that other suitably qualified foot and ankle specialists may have also elicited the same narratives from the participants.

*“I was referred to the physiotherapist … it took three years before seeing the right person (a podiatrist) and of course it was brilliant … he (the podiatrist) puts the finger on what the problem was” (Stanley).*

*“If I hadn't had seen Mr X (podiatrist) what would have happened? Would I have ended up having surgery and I think that would have been, you know, completely, well I mean unnecessary” (Mila).*

GPs are gatekeepers who co-ordinate access to health care treatment [12, 36]. Despite best intentions by GPs to make the most appropriate referral, this process can be complicated by constraints like cost, local policies, availability of resources or other factors [36]. A study [36] which investigated GPs referral behaviour, reported that GPs tend to refer patients to specialists within their own personal networks and not necessarily to the most appropriate practitioner. This type of referral behaviour may have an adverse impact the patient journey. This study demonstrates how important it is for patients to be referred to the most appropriately experienced practitioner(s) for their treatment as this can have a detrimental impact on the lived experience for patients with PTTD [34].

### Patient journey + loss

The chronicity of the journey imposed psychosocial and economic costs on participants. This included loss either through their social networks, taking time off work, or where previously activities or roles, which were taken for granted are now put into question because of PTTD [35, 37]. All participants experienced some form of loss through reduced activity or social limitation. Mila considered herself and active person however she had to limit going to concerts due to the instability and pain she felt when standing for long periods of time. Mark had to limit playing bowls, which was an important physical and social activity for him

*“ … having to stay at home and not do things … and be conscious … oh I can't do this because I can't stand up, like gig tickets and things like that … it just didn't feel stable” (Mila).*

*“I do play bowls. I don’t play as much now, because I have to stand all of the time … it’s not so easy anymore” (Mark).*

These important social activities that gave meaning to Mila and Mark’s lives, are now less accessible due to PTTD. They are experiencing a crumbling away of a former self without one of equal value taking its place and this represents loss [37]. This erosion and loss of the former self resulted in an undercurrent of stoicism which meant that patients would have some control over the experience of loss within their life worlds.

### Patient journey + stoicism

Stoicism can be defined as the endurance of pain or hardship without complaint [35]. Stoicism featured in all the participants interviews. Interestingly, British stoicism was also cited in a previous study in relation to a low uptake to surgery [25]. Some participants mentioned once or twice that they ‘carried on’ with their activities of daily life despite feeling pain or increased difficulty, while others mentioned this multiple times in their interviews.

*“Stupidly, I just carried on going to work and going to Zumba” (Mila).*

*“I just got on with it” (Faye).*

This endurance of pain without complaint is a psychological mechanism for participants to conceal the loss of their former productive selves because of the functional restrictions imposed on them by PTTD. Stoic behaviour may also protect participants from the stigma linked to becoming a burden to family, friends or colleagues [37]. This stoic behaviour may protect the participants psychologically by concealing the erosion of their former self from others. However, it may have a deleterious physiological impact on the posterior tibial tendon through inappropriate tissue stress [38] thus leading to prolonged symptoms. Other shared experiences were uncovered in the form of treatment burden which will now be discussed.

### Treatment burden

Treatment burden refers to the workload of healthcare and its impact on patient function and well-being [39]. The workload of health care is the burden associated with maintaining health in the context of chronic injuries which can include hospital visits, medical tests, treatment management and lifestyle changes [40]. Treatment fatigue may also develop as a result of treatment burden [41]. Tables 4 and 5 summarises the treatments undertaken by participants prior to and after presenting at the private podiatry clinic.

**Table 4:** Treatments undertaken prior to self-referral to private podiatry clinic

Participant (pseudonym)	Orthoses	Braces	Exercises	Steroid injection	Lifestyle changes
Mark	No	No	Yes	No	No
Faye	OTS	No	No	No	No
Mila	No	No	Yes	No	No
Stanley	No	No	Yes	No	No
Christopher	OTS	Airlift	No	Yes	Yes

Abbreviation: Off the shelf (OTS)

**Table 5:** Treatments undertaken after presentation to private podiatry clinic

Participant (pseudonym)	Orthoses	Braces	Exercises	Steroid injection	Lifestyle changes
Mark	Custom	No	Yes	No	Yes
Faye	Custom	No	Yes	No	Yes
Mila	OTS	Active Ankle	Yes	Yes	Yes
Stanley	Custom	Active Ankle	Yes	Yes	Yes
Christopher	Custom	Active	Yes	No	Yes

Abbreviation: Off the shelf (OTS)

Conservative treatment for PTTD usually involves two or three concurrent treatments which include exercises, FO and/or ankle bracing, onward referral for imaging, injections and lifestyle changes [15]. Lifestyle changes may include footwear change or activity modification. A significant amount of time and effort is required from the patient when managing PTTD through attending appointments, implementing treatments and lifestyle changes. Treatment burden and treatment fatigue may be significant clinical issues for patients with PTTD because it increases a patient’s workload of healthcare which may result in non-concordance to treatment. There are no clear guidelines for staging treatments for PTTD, unlike other common MSK foot and ankle conditions like plantar fasciitis or Achilles tendinopathy which are evidenced within NHS clinical knowledge summaries [14]. This lack of guidance may contribute to an increase in treatment burden because practioners don’t have the same level of information on which to guide their clinical decisions regarding effective conservative management.

Most of the participants in this cohort experienced some form of treatment burden / fatigue with respect to the prescribed exercises and orthoses. This had psychological consequences when the orthoses or exercises were not having a positive affect in reducing pain or improving function. This led to doubt and made the participants question the effectiveness of the treatments prescribed. Therefore, treatment burden is an important factor during management and may offer one explanation as to why this condition difficult to treat [40].

Although Mila was engaged in rehabilitation, the insinuation of treatment fatigue was present in her discourse because of the negligible benefit physiotherapy was having on reducing her pain [41].

*“I felt awful, yeah I mean I was doing them (exercises) it was painful, and I was quite regimented, I was like I've got to do the physio because I've got to make it better … it had been going on a really long time it was just getting worse” (Mila).*

Treatment fatigue also resulted in some participants either not doing the exercises or changing their exercises. This was due to secondary issues that were caused by the exercises given.

*“If I am honest I found them a bit tiresome (exercises)...they are difficult to fit into my day, so I did not do them all of the time” (Mark).*

*“I did the exercises, but I started having trouble with my hip, so I started swimming and the aches and pains disappeared, quite frankly I just carry on with that now” (Christopher).*

When patients stop their prescribed exercises, they are often labelled as ‘non-concordant’. This can cause some tension between the practitioner and the patient, which makes PTTD difficult to treat [35]. However, these behaviours can be explained through motivational control theories of fatigue and burden, whereby if there are no tangible benefits seen through the application of effort, like symptom reduction, then the patient is likely to decrease their adherence to a treatment or allocate effort elsewhere [41, 42].

This evidence highlights treatment burden and its impact on concordance and well-being during treatment. Ensuring treatment burden is kept to a minimum and recognising that treatment burden is likely to factor during conservative treatment for PTTD and mitigating its impact may help to improve concordance and the lived experience.

### Treatment burden + FO

FO are prescribed in most cases when patients are diagnosed with PTTD. FO address the pathophysiological aspects of PTTD as their aims are to reduce tissue stress on the PTT and, its associated structures, while improving foot, ankle and lower limb function [43].

All participants were prescribed and used FO and most of the participants understood and accepted the functional benefit of FO in relation to their treatment. In clinical practice it is not unusual to modify FO as they may not be tolerated from first use. Recent research [44] suggests that it is common place to modify orthoses in clinical practice. This research reports that the reason for this is because a systematic process in making orthoses may be lacking [44, 45]. Authors report that while there is evidence that a change in geometry of a device affects foot kinematics and kinetics, there is no evidence that making multiple adjustments to an orthotic results in a proportional change in clinical outcome and this questions the need for routinely adjusting orthoses [44].

Modifying FO increased treatment burden and introduced doubt regarding the efficacy of the device for participants in this study.

*“I know I keep saying it, but it’s a sometimes thing. They help some of the time but not all the time...(the orthoses) … I have to come here to have them changed” (Mark).*

*“Not sure if it’s quite the right orthotic even after it was changed … perhaps I should feel more support than I do” (Faye).*

Considering this evidence and the effects of treatment burden in PTTD, clinicians should produce the most appropriate device the first time where possible. Clinicians should explain the rationale behind changes or manage patients’ expectations regarding FO modifications. This may help to reduce the treatment burden and doubt for those with PTTD when FO are prescribed.

### Treatment burden + imaging and steroid injections

All participants were referred for some form of medical imaging prior to presenting at the private podiatry clinic. However, while beneficial in terms of diagnosis, the participants spoke of the negative lived experiences associated with some forms of imaging and steroid injections.

Typically, the results of the imaging were not explained in language that participants could understand. When they were offered the opportunity to view the images it did not help with their understanding of the imaging or the pathology.

*“I remember he showed me the debris on MRI … I think he showed the tear as well, I didn't really know what I was looking for, so I was just like OK that's the tear you know, I don't know what it looked like normally so … ”(Mila).*

When Christopher went for his ultrasound in the NHS, he said that he felt his foot was treated like a separate entity from himself.

*“I wasn’t even part of the conversation, you know, I felt that if I could have sent my foot along to be ultra-sounded (sic), without me there, it would have been more convenient for everybody” (Christopher).*

Three of the participants had steroid injections and they all experienced fear of the injection or the prospect of possible tendon rupture.

*“The needle length is absolutely enormous … and it was really painful as well and I wasn't prepared for that” (Mila).*

*“I was losing confidence in this chap and slightly annoyed by his arrogance saying no, no, no that hardly ever happened (tendon rupture). I went through with it (steroid injection) and I walked away from there wondering if at any moment my ankle was going to give out and rupture” (Christopher).*

Most of the participants had MRI before having an ultrasound and the participants found the MRI machine very imposing. There was also a long wait in obtaining results for all participants post MRI. A recent study reports that high resolution ultrasound is slightly more accurate than 3 T MRI in the diagnosis of PTTD [46]. Ultrasound should be the first line in imaging when PTTD is suspected, moreover, results / opinion can usually be given immediately, and it is not as imposing as an MRI. However, improvements in explaining imaging and the risks of tendon rupture when therapeutic injections are given is also important. These changes in practice may improve the lived experience of imaging for patient’s, thus reducing treatment burden. More must be done to make ultrasound imaging available for the assessment and diagnosis of PTTD. This can be achieved through training and by the ratification of national guidelines recommending ultrasound imaging as a gold standard in the diagnosis of PTTD [12, 46].

### Self-concept + image in relation to braces and footwear

Self-concept can be defined as the organisation of attributes of the self that have become constant and require self-validation on a daily basis [37]. Essentially, when self-image is not compatible with an individual’s attributes of self because of illness and treatments undertaken (i.e. wearing braces or recommended footwear), it can result in an erosion of positive self-image leading to a change in self-concept [37]. This change in self-concept may have an impact on concordance with treatment or result in barriers to change.

### Self-concept + bracing

Ankle bracing is a therapeutic, functionally corrective intervention in the conservative treatment for PTTD [41]. Qualitative studies have suggested that bracing may be beneficial for PTTD in reducing pain and improving function [16, 47, 48]. However, interestingly, despite reported improvements in function and pain in these studies, self-reported outcomes do not always improve [48].

Braces are difficult to conceal compared to FOs because they are not wholly cloaked within shoes. Wearing a brace also limits clothing and shoe choice which may have an impact on identity, self-concept and cosmesis [35]. Cosmesis refers to the degree in which the user feels attractive and socially acceptable [43]. Many of the ankle braces used in clinical practice for PTTD are bulk manufactured and designs are based on mechanical correction and not on the individual [43]. They are difficult to conceal for some patients and are not based on individual cosmesis and therefore may not provide self-the validation required within the framework of self-concept.

The discourse within this cohort when elaborating on their lived experiences with braces was littered with metaphor and symbolism. This is in stark contrast when participants spoke about FO where the narrative was quite technical and grounded in an understanding that reflected how the device would impact on their function.

Mila wore her brace for several months, even when her symptoms had improved. The brace limited Mila’s clothing choice, but she felt dependant on it. Dependency was also a feature in previous research [27]. To her the brace represented the strength which she had lost due to PTTD and she used metaphor to explain this.

*“I was like Iron Man … I found it a bit of a lifeline in fact the physio had to really wean me off it” (Mila).*

Christopher also spoke to the benefit of the brace in terms of support, however during the interviews he referred to the brace as being ugly or a thing.

*(getting a brace) … “This was the best things (sic) they ever did, immediately wearing the splint, that ugly one … I immediately felt like my ankle was being supported, held into place” (Christopher).*

Mark’s experience with the brace was interesting in that his discourse revealed hints of stigma.

*“Wearing a brace is like using a stick it is just not me’. It is better to get your aches and pains away with exercises rather than a brace because you can become dependent on it. I will try it, but I am not going to like it” (Mark).*

Wearing a brace is sign of disability, which carries with it stigma (43). Therefore, concordance with braces may be reduced because they are not as acceptable due to non-validation of self-concept or perceived as a negative treatment, compared to FO. This evidence may partially explain the reported poor satisfaction for participants in previous studies, despite improvements in pain and function [25].

### Self-concept + footwear

Footwear is an expression and extension of our self-concept and can convey messages about gender, taste and character [49, 50]. Footwear can be a repository for memories and certain types of shoes can represent significance and meanings in our lives and this can evoke emotion [50].

Christopher spoke of his memories regarding footwear and the inheritance of his father’s feet. This legacy of a certain foot type which he described as rubbish redefined the purpose of footwear for Christopher which meant he was limited in shoe choice.

*“So I assumed as I have my father’s rubbish feet and I would be wearing them the rest of my life (boots) … I remember when I was told that I had problems with my feet, it was from that moment that footwear wasn’t just something to stop your feet from getting wet, it’s sort of something I need to keep walking” (Christopher).*

In PTTD footwear change is usually recommended if current footwear is not suitable in terms of stability. It is common practice to recommend stability footwear / trainers or ankle style boots which aim to ‘support the foot’. Patients are asked to wear these most of the day to help reduce the symptoms associated with PTTD.

*“I have to wear these hideous shoes (trainers), but I don’t like it...it’s not very elegant” (Faye).*

*“I did have a little cry inside when I looked at what was recommended (trainers and sandals), I was just like, ergh” (sic) (Mila).*

*“I am not a trainer’s man and I will never be, they are just not me’. I don’t think they are very smart” (Mark).*

For Faye, wearing trainers did not align with her self-concept of elegance and her discourse hints at her inability to be feminine in trainers. Faye’s notion of elegance may be a self-concept linked to what Connell [44] has termed as ‘emphasized femininity where gendered embodiment emerges through negativity or the down-playing of body attributes that are shared by men [51]. For Mila, although throughout her interview she commended the benefits of trainers, the suggestion in her discourse was that other footwear recommended did not align with her self-concept.

Mark struggled with the concept of wearing trainers as he had always worn smart shoes until he was diagnosed with PTTD. When he talked about wearing trainers they did not align at all with his self-image. This caused a delay in him changing his footwear. As a result, he did not tolerate the orthoses as well as the other participants and he had the highest number of modifications to his device in the cohort.

Wearing footwear that causes conflict within a person’s self-concept and does not allow for self-validation, increases the loss of identity which influences the lived experience and transpires in a resistance to change in footwear [50]. Therefore, it is not surprising that patients struggle with changing their footwear in order to expedite treatment, because what is recommended may not always align with a patient’s self-concept and image [43]. Recognising the influence of self-concept and addressing this with the patient can help them to make a more informed decision about the benefits of the suggested footwear because wearing non-supportive footwear may render a less beneficial outcome in terms recovery.

Understanding the meaning of footwear and braces through the framework of self-concept can help to establish a meaningful dialogue with the patient about their footwear and may expose reasons behind potential resistance to change [50]. Having conversations with patients about their footwear and integrating the management of self-concept into treatment may help improve outcomes and the lived experience for patients with PTTD.

### Limitations and further research

Although this research has highlighted novel ideas regarding the lived experience for patients with PTTD, it is not without limitations. When reflecting on the interview process, interviews were held in the clinic where patients had been treated and this may have influenced the patient’s responses to questions and thus created bias in the data.

Due to the small sample size and sampling bias (purposive sampling), the findings cannot be extrapolated to the general population. Moreover, these experiences may not be representative of all patients with PTTD. However, this research addresses a gap in the literature for PTTD regarding the lived experience of treatment.

The experiences of the researcher also be seen to impose bias on the interpretation of the data. However, the role of reflexivity and the stance of the researcher should be present in within the interpretation of the data and this is recognised as important in IPA. Given the novel results of this research in relation to treatment burden, future research focusing on how this could be mitigated is indicated to help improve the lived experience for patients with PTTD.

## Conclusion

This study has investigated the lived experience of a group of patient’s receiving conservative treatments who have PTTD using a methodological process which is under-researched in current evidence. This study addresses a gap in the literature as it elucidates the lived experiences of treatment for patients with PTTD, which was previously unknown.

The scope of this study does not allow for further exploration of the issues around the paucity of guidelines for PTTD. The results however show how a lack of evidence-based guidelines can impact on the patient journey both through inappropriate referral and through inappropriate staging of treatments which may result in continued symptoms for some patients with PTTD. More research needs to be undertaken to address this issue, with integration of new guidance into national guidelines so practitioners are better informed about the management of this MSK condition. Should there be major developments in national guidelines, it would be worth repeating this study to audit any change in the lived experience of treatment for patients with PTTD.

The feature of loss is established within this research due to the impact PTTD has on physical and psychosocial function which influenced the lived experience of treatment. This study uncovered novel concepts such as treatment burden for patients with PTTD which can impact on well-being and thus the lived experience of treatment.

Considering these novel aspects while managing and treating PTTD may help improve the lived experience for those with PTTD. The hope is that this research will encourage practitioners who treat PTTD to consider the psychosocial factors that have been identified in this study. However, it is clear more research needs to be undertaken to help improve the lived experience for this group of patients.

## Supplementary information

**Additional file 1.** The interview guide: What are the lived experiences of conservative treatment for patients with Posterior Tibial Tendon Dysfunction.

## Data Availability

All consent forms and full transcripts will be held in the a co-authors office (CMR) for up to 10 years in a locked cabinet and these are stored and secured under the requirements of the GDPR, 2018.

## Abbreviations

AFO: Ankle foot orthoses
FO: Foot orthoses
IPA: Interpretative phenomenological analysis
MSK: Musculoskeletal
PTTD: Posterior tibial tendon dysfunction
QoL: Quality of Life
UCBL: University of California Biomechanics Lab
