# Supplementary material for: “I need somebody who knows about feet” a qualitative study investigating the lived experiences of conservative treatment for patients with posterior tibial tendon dysfunction
Source: J Foot Ankle Res. 2019 Nov 7;12:51. doi: 10.1186/s13047-019-0360-z (PMC6839224; doi:10.1186/s13047-019-0360-z)
Supplement: Supplementary file 1 — Additional file 1. The interview guide: What are the lived experiences of conservative treatment for patients with Posterior Tibial Tendon Dysfunction. [file 13047_2019_360_MOESM1_ESM.docx]

**APPENDIX 1**

The interview guide: What are the lived experiences of conservative treatment for patients with Posterior Tibial Tendon Dysfunction.

| Section Steps | Details |
| --- | --- |
|  |  |
| **Opening (5 mins)** | - Refreshments provided, researcher introduced, role explained during the session to establish comfortable facilitation of the interview. - Handout of information form with informed consent given with presentation of the study goals. - Participants made aware that the session will be recorded and anonymized. |
|  | - Information recorded on gender, age and a summary of diagnosis. |
| **Potential topics** | - Introduction prompt: You have been diagnosed and treated for this foot condition known as PTTD. We are going to talk today about your lived experiences in relation to the treatments/management you have had / are receiving. - Prompt#1: Tell me what conservative (non-surgical) treatments you received for this condition and your experiences of these treatments in your daily life? |
| **Footwear** | - Question: Tell me about the time you were advised about footwear / changing your footwear as part of your treatment your experiences regarding changing your footwear. - Prompt#1: What adjustments in your life have you had to make to accommodate for the recommended footwear? - Prompt#2: Tell me about or describe the experience of having to change your footwear to help enable / facilitate treatment?   Prompt#3: Was the experience of changing your footwear challenging in terms of your daily life (e.g. work / image / activities). |
| **Foot orthoses** | - Question: Could you please tell me about your experiences regarding the foot orthoses prescribed as part of treatment? - Prompt #1: Describe your experiences with wearing the foot orthoses, are they comfortable, improving the pain, or uncomfortable, not improving the pain, difficult to fit in all shoes, cumbersome? What specifically is it about the foot orthoses that led to these experiences? Can you be specific and detailed about a time when you experienced problems or benefits from using the foot orthoses? - Prompt #2: Can you tell me about the time you were given orthoses and the experience between yourself and the dispensing clinician? |
|  |  |
| **Ritchie Brace**  **(brace participants)** | - Question: You have been prescribed with an ankle brace. Tell me about your experiences of wearing the brace? - Prompt#1: Can you describe how the experience of wearing the brace has influenced your daily life? - Prompt #2: Has wearing the brace been a beneficial experience or has it been negative in terms of it improving your activities in your daily life? - Prompt#3: How does wearing this brace impact on your choice of clothing / activities and what are your experiences in dealing with these problems? |
| **Diagnostic imaging**  **& therapeutic injections** | - Intro prompt: You were referred on for imaging / imaging and injection. - Question: Tell me about your experience in relation to being referred for imaging or injections?   Prompt #1: What was your experience of being told the results of the imaging and did it help you understand this condition? If you were not told or informed of the results, did your experience of this have an impact on your treatment?   - Prompt #2: If you received an injection as part of treatment, tell me about your experience of this? |
| Physical therapy | - Question. Tell me about your experiences in relation to doing the exercises? - Prompt #1: What is your experience of the benefit from exercises? - Prompt #2: Have you encountered any problems regarding the exercises? - Prompt #3: What is your experience of having the exercises explained to you? |

Closing Do you have any further experiences to share regarding your experience?

Thank you

(Version 3, Jan 2017)
